# Clock Gene Expression in Eel Retina and Hypothalamus: Response to Photoperiod and Moonlight

**DOI:** 10.1002/jez.2870

**Published:** 2024-10-07

**Authors:** Ji‐Yeon Hyeon, Jun‐Hwan Byun, Byeong‐Hoon Kim, Sachithra Amarin Hettiarachchi, Jeonghoon Han, Young‐Ung Choi, Choong‐Hwan Noh, Yuki Takeuchi, Soo‐Youn Choi, Jong‐Eun Park, Sung‐Pyo Hur

**Affiliations:** ^1^ Marine Biotechnology & Bioresource Research Department Korea Institute of Ocean Science & Technology Busan Republic of Korea; ^2^ Department of Fisheries Biology, College of Fisheries Sciences Pukyong National University Busan Republic of Korea; ^3^ Education & Research Group for Future Strategy of Aquatic Life Industry Jeju National University Jeju Republic of Korea; ^4^ Division of Polar Life Science Korea Polar Research Institute Incheon Republic of Korea; ^5^ Department of Ocean Science University of Science and Technology Daejeon Republic of Korea; ^6^ Developmental Neurobiology Unit Okinawa Institute of Science and Technology Kunigami‐gun Okinawa Japan; ^7^ Department of Biology Jeju National University Jeju Republic of Korea; ^8^ Department of Animal Biotechnology, Faculty of Biotechnology, College of Applied Life Sciences Jeju National University Jeju Republic of Korea; ^9^ Department of Marine Life Science Jeju National University Jeju Republic of Korea

**Keywords:** circadian rhythm, clock gene, eel migration, Japanese eel, moonlight

## Abstract

Assessment of the clock genes, Period (*Per*) 1, *Per2*, *Per3*, and Cryptochrome (*Cry*) 2, *Cry3*, and *Cry4*, can help better understand eel spawning ecology. In this study, the circadian rhythm and moonlight effects of these clock genes in the eel retina and hypothalamus were analyzed. We examined clock gene expression patterns under 12 h light:12 h darkness (12L12D), constant darkness (DD), and constant light (LL) conditions; under short photoperiod (SP; 9L15D) and long photoperiod (LP; 15L9D), and during the new moon (NM) and full moon in male eels. *Per2* expression increased after sunrise, *Cry2*, and *Cry4* expression increased around sunset, and *Per1*, *Per3*, and *Cry3* expression increased before sunrise. Under SP conditions, oscillations of retinal *Per3* and *Cry4*, which did not occur under LP conditions, were generated. In addition, retinal *Cry4* oscillation was generated under NM conditions. These results suggest that the retina of the eel may play an important role in regulating circadian rhythm, and migration is initiated by the synchronization of clock genes by moonlight, suggesting that photic signals are closely related to the migratory activity of the eel.

## Research Highlights

- Clock gene expression was analyzed under various conditions in the retina and hypothalamus of eel.
- *Per2* mRNA expression peaked during the photophase, *Cry2* mRNA expression peaked before the onset of the scotophase, and *Cry4* mRNA expression peaked after the onset of the scotophase.
- *Per1*, *Per3*, and *Cry3* mRNA expression peaked before the onset of the photophase, inducing oscillations.
- Under short photoperiod condition, oscillations of *Cry4* and *Per3* were induced, whereas under the new moon condition, oscillations of *Cry4* were induced.

## 1 Introduction

Circadian rhythms are endogenous rhythms with a period of approximately 24 h and found virtually in all light‐sensitive organisms (Paranjpe and Sharma 2005). Most organisms have an internal clock and their circadian rhythms exhibit certain basic features. In general, the circadian rhythm is an oscillation that operates at a cycle of approximately 24 h, and recognition and synchronization of daily behavior and homeostasis occur depending on external light stimuli. Although such oscillations are generated and controlled at the molecular level, it may influence several aspects of organisms, including physiology and behavior. Moreover, circadian clocks have been shown to enhance the innate ability of several organisms to adapt and survive under changes in environmental conditions, such as light and climate (Paranjpe and Sharma 2005; Vaze and Sharma 2013). Particularly, in vertebrates, predictable environmental temporal events, such as the 24‐h light/dark (LD) cycle, are considered the most important external zeitgebers for circadian systems (Hastings, O'Neill, and Maywood 2007). Further, the regulation of the circadian clock in animals other than mammals is closely linked to the external LD cycle signaling through the optical input pathway and biological clock synchronization via the endogenous optical input pathway. In this regard, the circadian system in fish comprises other elements by which light is sensed by the organism and converted into neuronal or endocrine hormonal signals, and circadian rhythms regulated by light signal input are well known to affect fish reproduction and growth rhythm (Falcón et al. 2010). The circadian rhythm is created and maintained by a transcription‐translational feedback loop that is automatically regulated by the interaction of clock genes (Dunlap 1999; Pando and Sassone‐Corsi 2002). This regulation is of two types: transcriptional activation and repression. The heterodimerization of the transcription factors, circadian locomotor output cycle kaput (CLOCK), and brain and muscle aryl hydrocarbon receptor nuclear translocator‐like protein1 (BMAL1), which act as positive regulators, facilitates binding to the E‐box in the promoters of the Period (*Per*) and Cryptochrome (*Cry*) genes, thus stimulating overall transcription. Studies have shown that clock genes, such as *Per* and *Cry*, are involved in the regulation of behavioral process rhythms and reproduction in many life forms (Chen et al. 2017). The lunar cycle drives moonlight intensity, tidal amplitude, and periodic changes in the geomagnetic field. These factors can affect reproduction in organisms. Marine organisms that are affected by the lunar cycle have evolved survival strategies, such as spawning mainly at night to avoid the effects of tide in coastal waters. Changes in moonlight intensity affect gonadal development and gamete release during a specific lunar phase (Takemura, Rahman, and Park 2010). Previous studies have reported that *Per* and *Cry* genes may respond to lunar periodicity and have suggested that moonlight is involved in the reproduction of fish (Ikegami, Takeuchi, and Takemura 2014). Therefore, studies on physiological changes, such as reproductive activity by moonlight and lunar phases, expression of clock genes, *Per* and *Cry*, and changes in melatonin secretion in various marine species, can improve our understanding of lunar‐biospheric relationships. The periodic change in moonlight also affects the spawning migration of some teleosts. In particular, teleost eels are well known as migratory fish that migrate long distances at specific periods for spawning. These functional‐dependent behaviors have been reported to be highly associated with and dependent on the influence of moonlight (Miyai et al. 2004; Tesch 1978; Tsukamoto 2006). For example, the European eel, *Anguilla anguilla*, swims from the bottom with cold‐water layers to the top with warm water layers of the ocean in the presence or absence of moonlight during the migratory process or begins downstream migration for spawning at the end of the year (Miyai et al. 2004). Eels are more sensitive to moonlight than to temperature during spawning migration (Tesch 1978). Based on these reports, it can be assumed that moonlight is closely correlated with reproductive behavior in eels. However, although it is known that moonlight is related to the reproduction of eels, the effects of moonlight on physiological and biochemical processes, particularly on the circadian mechanism, in eels still require further studies.

Japanese eel (*Anguilla japonica*) has become a very economically and socially important culture species in East Asia. Due to a rapid decline in its natural population, there have been various attempts in East Asia to focus on artificial seed production (Hamidoghli et al. 2019). However, artificial seed production has not been possible due to problems, such as artificial reproduction induction and feed development.

To date, although the spawning area (West Mariana Ridge) of *A. japonica* has been revealed (Kimura, Inoue, and Sugimoto 2001; Aoyama et al. 2014), many aspects of the ecology and physiology of eel's migratory process have remained a mystery. Therefore, to comprehend the enigmatic ecological and physiological mechanisms of eels, one must understand various environmental aspects of marine biology. In particular, to understand how light, a factor regulating circadian rhythms, affects the ecological and physiological aspects of this species, one must first understand its role among the various environmental factors that induce physiological activity.

In this study, the *Per* and *Cry* genes were identified from the RNA‐seq data of *A. japonica*. Using real‐time quantitative reverse‐transcription polymerase chain reaction (qPCR), we investigated the light‐dependence of the *Per* and *Cry* genes through analysis of transcriptional levels of the core loop to understand the working principle of the little‐known endocrine concepts of time, circadian rhythm, and seasonality in the eel retina and hypothalamus. This study will provide a better understanding of how moonlight affects the relevant molecular response of the circadian system in the Japanese eel.

## 2 Materials and Methods

### 2.1 Animals and Maintenance

The Japanese yellow eels (*n* = 234, 2 years old) used in the study were male (body weight: 280–405 g) and obtained from a commercial source in Gwangju Prefecture, South Korea. They were kept in circular tanks (one‐metric ton capacity) with running fresh water and maintained at a temperature of 20 ± 1°C under an LD cycle (light on at 07:00 h and light off at 19:00 h, 600 lx, PPFD = 10.0 µmol m^−2^ s^−1^, *λ*p = 545 nm) with white light‐emitting diode (LED) light (KRGB3, SS Light, Co., Seoul, South Korea). The eels were not fed during the experiment, and all procedures were approved by the Animal Care and Use Committee of the Korea Institute of Ocean Science and Technology (KIOST 2021‐0001).

#### 2.1.1 Experiment 1: Assessing the Expression of Clock Genes in the Retina and Hypothalamus Under the LD, Constant Light (LL), and Constant Dark (DD) Conditions

**Figure 1:**
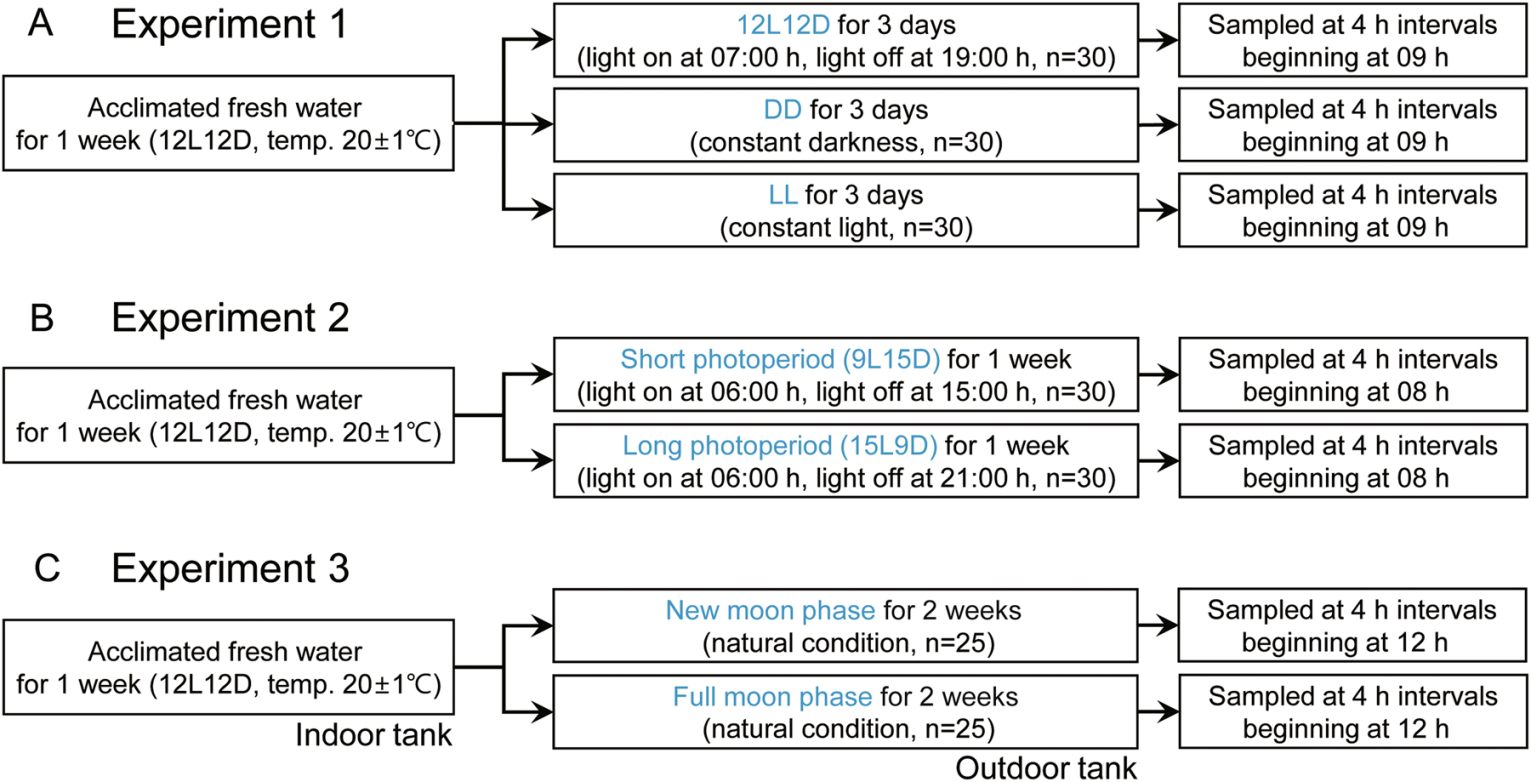
Experimental design in this study. Before all experiments, eels were acclimated to freshwater for 1 week. (A) In Experiment 1, the eels were reared under 12L12D, DD, and LL conditions for 3 days each. (B) In Experiment 2, eels were exposed to 9L15D and 15L9D conditions for 1 week each. (C) In Experiment 3, fish were transferred to outdoor tanks and reared for 2 weeks. All eels were sampled every 4 h over a 24‐h period.

To evaluate the daily and circadian fluctuations in transcript levels of clock genes in the retina and hypothalamus, 30 eels per tank were housed in three freshwater tanks with a one‐metric ton capacity. The tanks did not have any fish shelters and were maintained under the 12L12D condition at a water temperature of 20 ± 1°C. After 1 week of acclimation, the eels were reared for 3 days either under LL or LD conditions with a light intensity of approximately 600 lx (10.0 µmol m^−2^ s^−1^, *λ*p = 545 nm) at the water surface with a water temperature of 20 ± 1°C. Additionally, for comparison, 30 eels were also kept under the DD condition for 3 days. To collect tissue samples, the eels were anesthetized using 150 mg L^−1^ of MS‐222 (Sigma‐Aldrich, St. Louis, MO, USA), and every 4 h, starting at 2 h after light onset, five eels from one tank were decapitated at each sampling time (Figure 1A).

#### 2.1.2 Experiment 2: Assessing the Expression of Clock Genes in the Retina and Hypothalamus Under Short Photoperiod (SP) and Long Photoperiod (LP) Conditions

To investigate the impact of changes in photoperiod on the levels of clock gene transcripts in the retina and hypothalamus, 36 eels were placed in two tanks (one metric ton capacity) under LD conditions with a water temperature of 20 ± 1°C for 1 week to acclimate. After the acclimation period, the photoperiod in the tanks was altered to a SP condition (9L15D, lights on at 06:00 h and off at 17:00 h) or a LP condition (15L9D, lights on at 06:00 h and off at 21:00 h) for 1 week. At 4‐h intervals beginning at 2 h after light onset, five eels were anesthetized and decapitated from each tank for sampling (Figure 1B).

#### 2.1.3 Experiment 3: Assessing the Expression of Clock Genes in the Retina and Hypothalamus Under New Moon (NM) and Full Moon (FM) Conditions

We conducted a comparison of clock gene transcript levels during the NM and FM periods. A total of 25 eels were placed in an indoor tank with recirculating and aerated fresh water under LD conditions at 20 ± 1°C. After a 1‐week acclimation period, the eels were transferred to two outdoor acrylic tanks (three‐metric ton capacity). The rearing tanks were exposed to natural photoperiod conditions (approximately 11L13D) with recirculating water (20 ± 1°C) for 2 weeks but without a cover, until the end of the experiment. Samples were collected during the NM (*n* = 25) and FM (*n* = 25) periods. On each sampling day, the eels were anesthetized and euthanized by decapitation following the guidelines mentioned above at 4‐h intervals beginning at 12:00 h. After weighing, the left eye and hypothalamus were immediately collected, frozen in liquid nitrogen, and stored at −80°C until analysis. All sample collections were conducted under dim‐light conditions (1.5 lx, 0.0 µmol m^−2^ s^−1^ at 670 nm) using a red‐light LED module (Figure 1C).

### 2.2 Total RNA Extraction and cDNA Synthesis

Total RNA was extracted from the retina and hypothalamus using RNAiso Plus (Takara Bio, Otsu, Japan), following the manufacturer's instructions. Isolated total RNA (1 μg) was treated with DNase I (Promega, Madison, WI, USA) at 37°C for 15 min to prevent contamination with genomic DNA. The total RNA quantity was measured using NanoDrop One C (Thermo Fisher Scientific, Waltham, MA, USA) at 260 and 280 nm, and samples with an A260:A280 ratio between 1.8 and 2.0 were used for cDNA synthesis. RNA from the retina and hypothalamus was mixed at the same concentration and volume for RNA sequencing (RNA‐seq). cDNA was synthesized using the Transcriptor First Strand cDNA Synthesis Kit, according to the manufacturer's protocol (Roche Diagnostics, Indianapolis, IN, USA).

### 2.3 cDNA Library Construction and Massively Parallel Sequencing

The Illumina TruSeq RNA Sample Preparation Kit v2 (catalog #RS‐122‐2001; Illumina, San Diego, CA, USA) was used to prepare RNA‐seq paired‐end libraries. Total RNA was obtained from the retina and hypothalamus, and genomic DNA was removed to prevent contamination. The quality and quantity of RNA were evaluated using the 2100 Bioanalyzer RNA 6000 NANO chip (Bio‐Rad, Hercules, CA, USA). High‐quality total RNA from six individuals’ retina and hypothalamus was pooled. To generate cDNA, mRNA was purified using poly (A) selection, chemically fragmented, and converted into single‐stranded cDNA with random hexamer priming. Subsequently, double‐stranded cDNA was generated by the second strand. To construct the library, blunt‐end cDNA fragments were produced from ds‐cDNA. The blunt ends were then modified by adding an A‐base, allowing for the ligation of sequencing adapters. After ligate size selection, adapter‐specific primers were used to amplify ligated cDNA fragments. The KAPA library quantification kit (Kapa Biosystems KK4854) was used to quantify the library, following the manufacturer's instructions. The Illumina Hiseq. 2000 platform was used to load each library, and throughput sequencing (read length 2 × 100) was carried out to ensure that each sample met the desired average sequencing depth.

### 2.4 Preprocessing and De Novo Reconstruction of Transcriptome

Trimmomatic (ver. 0.3.6, Bolger, Lohse, and Usadel 2014) was used to trim the low‐quality and adapter sequences from both the 5′ and 3′ ends of each read. Additionally, PRINSEQ lite (ver. 0.20.4, Schmieder and Edwards 2011) was used to remove low average quality reads (*Q* < 25). The cleaned raw reads from the retina and hypothalamus RNA were combined and mapped to the *A. anguilla* draft genome sequence (Ref) using tophat2 (ver.2.1.0, Kim et al. 2013). A de novo transcriptome reconstruction was then performed using genome‐guided Trinity (ver. 2.3.2, Grabherr et al. 2011) with the beam mapping results. To create a unigene set and remove redundant contigs, the assembled contigs were clustered and filtered using cd‐hit‐est with default parameters, which are 0.9 identity threshold (‐c) and 10‐word length (‐n) (CD‐HIT package, Li and Godzik 2006).

### 2.5 Phylogenetic and Expression Analysis for the Clock Genes Using RNA‐Seq Data

To investigate clock genes in the Japanese eel's assembled transcriptome sequences, the tBlastn program was utilized with *A. anguilla* clock gene protein sequences (*E*‐value < 0.01). ORF Finder (http://www.ncbi.nlm.nih.gov/gorf/gorf.html) was used to identify the ORF regions of Japanese eel clock gene candidates, and the presumed protein sequences were aligned with those of teleost clock gene family proteins. To construct a phylogenetic tree, the neighbor‐joining method in MEGA11 (Tamura, Stecher, and Kumar 2021) was used. Five hundred bootstrap repetitions were conducted, and the values are shown at the inner nodes. To quantify the expression levels of the identified clock family genes, cleaned reads were mapped onto reconstructed contigs using Bowtie2 (Langmead and Salzberg 2012), and the expression levels were estimated using Tigar2 (Nariai et al. 2014).

### 2.6 Real‐Time Quantitative PCR

Using the BioRad CFX96TM Real‐Time System (BioRad) and SYBR Green premix II (Takara Bio), the expression of each target gene was analyzed. Primers for qPCR (Table 1) were designed with Primer‐BLAST (http://www.ncbi.nlm.nih.gov/tools/primer-blast/). Each PCR mix consisted of 50% SYBR Premix, 0.2 μM of each forward and reverse primer, and 2 μL of cDNA template. The amplification conditions were as follows: initial denaturation for 1 min at 95°C, followed by 40 cycles of denaturation for 45 s at 95°C, and annealing and extension for 1 min at 60°C. The expression of each gene in each sample was normalized to that of the internal control *EF1α* gene.

**Table 1:** Primer sets for qPCR.

**Gene ID**	**Oligo ID**	**Sequence**	**Product size (bp)**
*EF1α* (MH020210)	Forward	5′–TCACCCTGGGAGTAAAGCAG–3′	222
	Reverse	5′–TCCATCCCTTGAACCAGGAC–3′	
*Per1* (LC616387)	Forward	5′–GCAGAAGGAGCTGATGAAGG–3′	224
	Reverse	5′–TGATGTTGTCCAGCTCTTCG–3′	
*Per2* (LC616388)	Forward	5′–CAAGGCGAAAACTCAGAAGG–3′	232
	Reverse	5′–GTCGATCTCCTCGATGGTGT–3′	
*Per3* (LC616389)	Forward	5′–CACGCTGATCCTGCCAGTAA–3′	356
	Reverse	5′–CTGGTCAGGGAGAACACCAC–3′	
*Cry2* (LC616385)	Forward	5′–CCCCTCACCTACAAGCGTTT–3′	286
	Reverse	5′–TGGCGTTCATTCTGGGTCTC–3′	
*Cry3* (LC616386)	Forward	5′–GGGTCAGCCAGCAGATGTAT–3′	177
	Reverse	5′–TCCAGGTTGTAGAGCGTGTG–3′	
*Cry4* (OR805365)	Forward	5′–ATTCCAAGCCTGGAGGATCT–3′	159
	Reverse	5′–CGGTAACAGGGAGTTTGGAA–3′	

### 2.7 Statistical Analysis

All statistical analyses were performed using the GraphPad Prism software (version 8.0). In experiment 1, the expression of the clock genes was compared using a one‐way analysis of variance (ANOVA), followed by Tukey's multiple‐comparisons test. In experiments 2, and 3, comparisons of clock gene expression levels between groups were performed by the Unpaired *t* test. All circadian oscillation patterns of the clock gene expression were subjected to cosinor analysis using CircWave v1.4 (http://www.euclock.org/results/item/circ-wave.html). CircWave employs a forward linear harmonic regression model to calculate the profile of a 24 h period, with *α* set at 0.05. The *p* values reported are the result of the *F*‐test from the software.

## 3 Results

### 3.1 RNA‐Seq Analysis

Expression of the clock genes in the retina and hypothalamus of Japanese eels was investigated using RNA‐seq. After adapter trimming and quality filtering, 150,898,925 paired‐end reads were used for de novo transcriptome reconstruction. As a result of cd‐hit est clustering, 313,671 contigs (N50 = 965) were identified. tBlastn analysis identified two main gene families, *Per* and *Cry*, in the hypothalamus through RNA‐seq.

**Figure 2:**
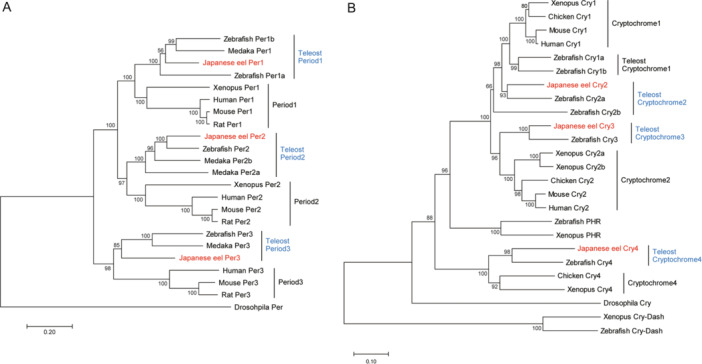
Phylogenetic tree of (A) *Per* and (B) *Cry* genes of vertebrates, including those of the Japanese eel. Five hundred bootstrap repetitions were performed and values are shown at the inner nodes. *Drosophila melanogaster* Per was used as an outgroup. The protein alignment was performed for the phylogenetic analysis by Clustal W. *Per* and *Cry* genes of Japanese eel, *Anguilla japonica*, are indicated in bold.

*Per1*, *‐2*, and *‐3* were 3173, 3657, and 1248 bp, respectively; *Per1*, *‐2*, and *‐3* comprised 1057, 1218, and 416 amino acids, respectively. Comparison of the amino acid sequence homology between the *Per* genes of the eel and other animal groups revealed that the homology of *Per1* was 59%–71% in fish, 57% in amphibians (*Xenopus laevis*), and 59%–61% in mammals (*Mus musculus*, *Rattus norvegicus*, and *Homo sapiens*). Low homology was observed in all other animal groups. The amino acid sequence homology of *Per2* was 61%–73% in fish, which was relatively high compared to that in other animal groups, but 47% in amphibians (*X*. *laevis*) and 49%–51% in mammals (*R*. *norvegicus*, *M*. *musculus*, and *H*. *sapiens*). It showed a relatively low homology. The amino acid sequence homology of *Per3* was 60%–64% in fish and 53%–55% in mammals (*M*. *musculus*, *R*. *norvegicus*, and *H*. *sapiens*), indicating low homology in all animal groups (Figure 2A).

The base sequences of three *Cry* genes in the hypothalamus of eels were also analyzed. *Cry2*, *‐3*, and *‐4* were 1857, 951, and 1002 bp, respectively, and *Cry2*, *−3*, and *4* comprised 618, 317, and 333 amino acids, respectively. Comparison of the amino acid sequence homology of the *Cry* genes in eels with that in other animal groups revealed that *Cry2* had a homology of 82%–91% in fish, 79% in amphibians (*X*. *laevis*), 80% in birds (*Gallus gallus*), and 81–82% in mammals (*M*. *musculus* and *H*. *sapiens*), indicating high homology in all animal groups. The amino acid sequence homology of *Cry3* was 84% in zebrafish (*Danio rerio*), indicating relatively high homology. In addition, the amino acid sequences of *Cry3* showed relatively high homology (73%) with the *Cry1* amino acid sequences of other animal groups (*Gallus gallus*, *M*. *musculus*, and *H*. *sapiens*). The amino acid sequence homology of *Cry4* was 72% in zebrafish (*D*. *rerio*), showing relatively high homology, but 65% in amphibians (*X*. *laevis*) and 65% in birds (*G*. *gallus*), indicating relatively low homology (Figure 2B). Most clock genes identified in this study showed relatively high identity to those of zebrafish.

### 3.2 Experiment 1: Assessing the Expression of Clock Genes in the Retina and Hypothalamus Under 12L12D (LD), LL, and DD Conditions

**Figure 3:**
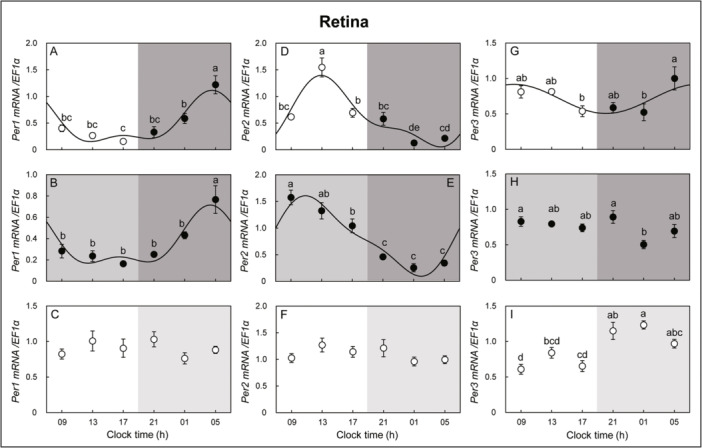
Cosinor analyses of *Per1*, *‐2*, and *‐3* gene expression levels in the retina of the eel for three days under (A, D, G) LD, (B, E, H) DD, and (C, F, I) LL conditions. The values are mean ± SEM (*n* = 5) of the normalized transcript levels of *Per1*, *‐2*, and *‐3* genes. White and black represent the light and photophase and scotophase, respectively. Significant differences between the means at each sampling time are indicated by different letters (*p* < 0.05).

The circadian oscillation patterns of clock genes of eels were investigated under LD, DD, and LL conditions. In the retina, *Per1* mRNA expression in the retina showed no circadian oscillation change in the LL condition but peaked at clock time (h) 05 in the LD and DD conditions, with a similar pattern of circadian oscillation. *Per2* mRNA expression showed similar oscillation patterns, with *Per2* mRNA expression peaking at 13 and 09 h in the LD and DD conditions, respectively, but no circadian oscillation change was observed in the LL condition. *Per3* mRNA expression showed circadian oscillation changes under LD conditions, but no oscillation changes were observed under DD and LL conditions (Figure 3). In the retina, the *Per1*, *‐2*, *Cry3*, and *‐4* mRNA expression oscillations showed similar patterns under LD and DD conditions.

**Figure 4:**
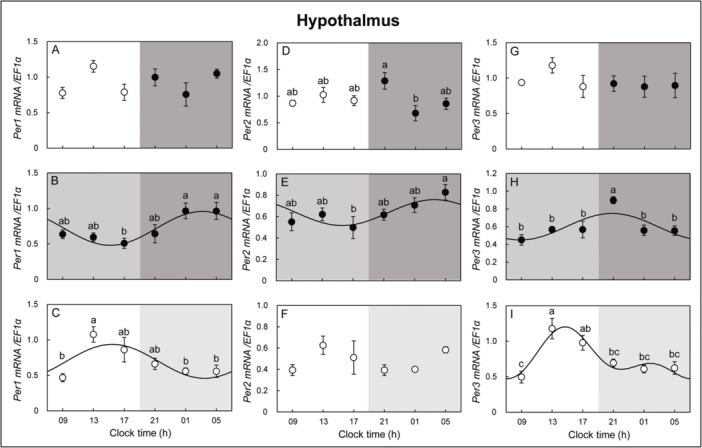
Cosinor analyses of *Per1*, *‐2*, and *‐3* gene expression levels in the hypothalamus of the eel for 3 days under (A, D, G) LD, (B, E, H) DD, and (C, F, I) LL conditions. The values are mean ± SEM (*n* = 5) of the normalized transcript levels of *Per1*, *‐2*, and *‐3* genes. White and black represent the light and photophase and scotophase, respectively. Significant differences between the means at each sampling time are indicated by different letters (*p* < 0.05).

**Figure 5:**
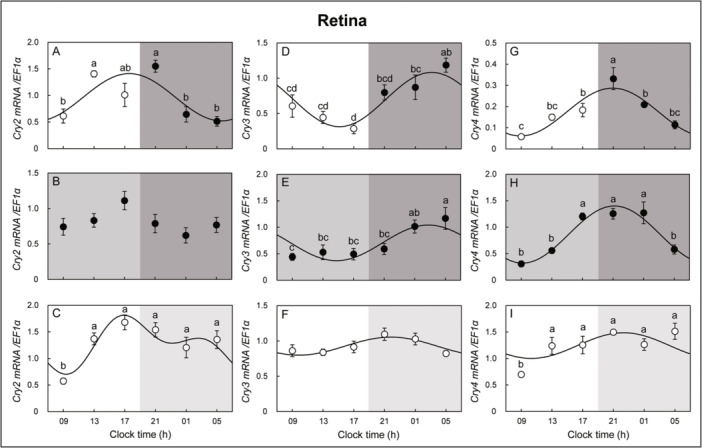
Cosinor analyses of *Cry2*, *‐3*, and *‐4* gene expression levels in the retina of the eel for 3 days under (A, D, G) LD, (B, E, H) DD, and (C, F, I) LL conditions. The values are mean ± SEM (*n* = 5) of the normalized transcript levels of *Cry2*, *‐3*, and *‐4* genes. White and black represent the light and photophase and scotophase, respectively. Significant differences between the means at each sampling time are indicated by different letters (*p* < 0.05).

**Figure 6:**
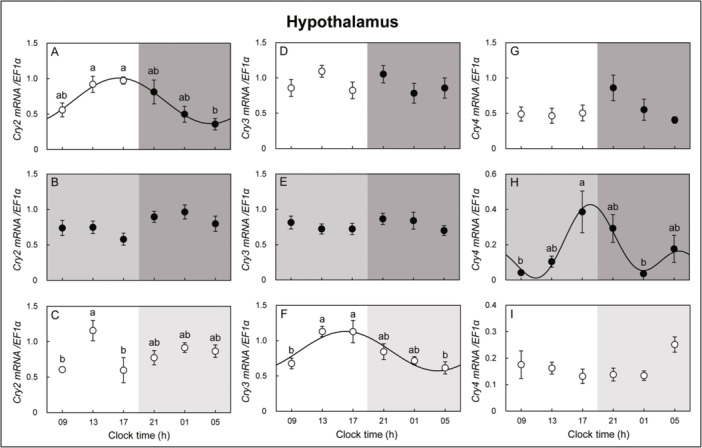
Cosinor analyses of *Cry2*, *‐3*, and *‐4* gene expression levels in the hypothalamus of the eel for 3 days under (A, D, G) LD, (B, E, H) DD, and (C, F, I) LL conditions. The values are mean ± SEM (*n* = 5) of the normalized transcript levels of *Cry2*, *‐3*, and *‐4* genes. White and black represent the light and photophase and scotophase, respectively. Significant differences between the means at each sampling time are indicated by different letters (*p* < 0.05).

In the hypothalamus, *Per1* mRNA expression showed an opposite circadian oscillation pattern under DD and LL conditions but no circadian oscillation changes under LD conditions. Circadian oscillation of *Per2* mRNA expression was observed in the DD condition but not in the LD and LL conditions. *Per3* mRNA expression showed circadian oscillation changes in DD and LL conditions but not in LD conditions (Figure 4). *Cry2* mRNA expression in the retina showed a circadian oscillation under LD and LL conditions but not under DD conditions. *Cry3* mRNA expression in the retina showed circadian oscillation changes under LD, DD, and LL conditions and peaked at 05 h under LD and DD conditions. Similar circadian oscillation patterns were observed under LD and DD conditions. *Cry4* mRNA expression in the retina showed a circadian oscillation change with a peak at 21 h under LD, DD, and LL conditions, and a similar circadian oscillation pattern was observed under LD and DD conditions (Figure 5). *Cry2* mRNA expression showed rhythmic changes in the hypothalamus under LD conditions, but no circadian oscillation was observed under DD and LL conditions. Hypothalamic *Cry3* mRNA expression did not show an oscillation under LD and DD conditions but showed circadian oscillation changes under LL conditions. *Cry4* mRNA expression in the hypothalamus did not show circadian oscillation changes under LD and LL conditions but showed a circadian oscillation under DD conditions (Figure 6).

### 3.3 Experiment 2: Assessing the Expression of Clock Genes in the Retina and Hypothalamus Under SP and LP Conditions

**Figure 7:**
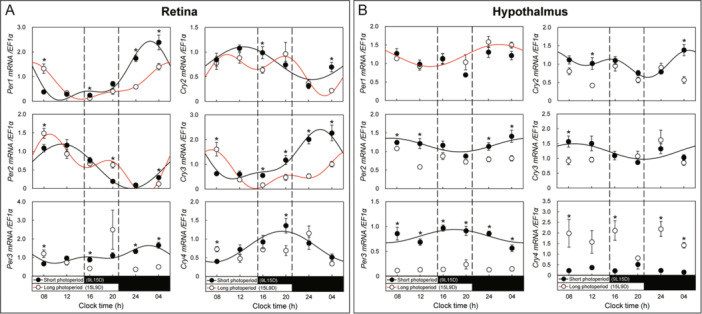
Cosinor analyses of clock gene expression levels in the (A) retina and (B) hypothalamus of the eel for 1 week during the short‐photoperiod (9L15D) and long‐photoperiod (15L9D). The values are mean ± SEM (*n* = 5) of the normalized transcript levels of clock genes. White and black represent the long photoperiod and short photoperiod, respectively. Significant differences between the means at each sampling time are indicated by different letters (*p* < 0.05).

Circadian rhythms of clock genes were investigated under SP and LP conditions in Japanese eels. In the retina, *Per1* mRNA expression showed an oscillation under both the SP and LP conditions, but the oscillation was delayed in the LP condition, and the SP condition oscillation was similar to that in the LD condition. *Per2* mRNA expression, under both the SP and LP conditions, showed an oscillation similar to that under the LD and DD conditions, but showed a slightly different oscillation pattern, with an additional peak at 20 h in the LP condition. *Per3* mRNA expression showed an oscillation only in the SP condition. *Cry2* mRNA expression showed oscillation under LP and SP conditions but showed a significant difference at 16 h with a different pattern of oscillation. *Cry3* mRNA expression peaked at 04 h under the SP condition and showed an oscillation similar to that in the LD condition. The oscillation was delayed in the LP condition, except at 08 h. *Cry4* mRNA expression showed an oscillation with a peak at 20 h under the SP condition in the retina (Figure 7A). In the retina, *Per1*, *‐2*, *Cry2*, *‐3*, and *‐4* mRNA expression oscillations showed similar patterns under SP and LD conditions.

In the hypothalamus, *Per1* mRNA expression showed an oscillation under the LP condition, but there was no significant difference in the mRNA expression levels under the SP condition in all sections. The oscillation pattern of Per2 mRNA expression in the hypothalamus under the SP condition was similar to that under the DD condition, and no oscillation was observed under the LP condition. Hypothalamic *Per3* mRNA showed higher expression in all sections under the SP condition than under the LP condition, and an oscillation was observed under the SP condition. In the hypothalamus, *Cry2* showed no oscillation under the LP condition and showed oscillation under the SP condition. Hypothalamic *Cry3* mRNA expression showed an oscillation under the SP condition. *Cry4* mRNA expression showed no oscillation in the hypothalamus under both the SP and LP conditions (Figure 7B).

### 3.4 Experiment 3: Assessing the Expression of Clock Genes in the Retina and Hypothalamus under NM and FM Conditions

**Figure 8:**
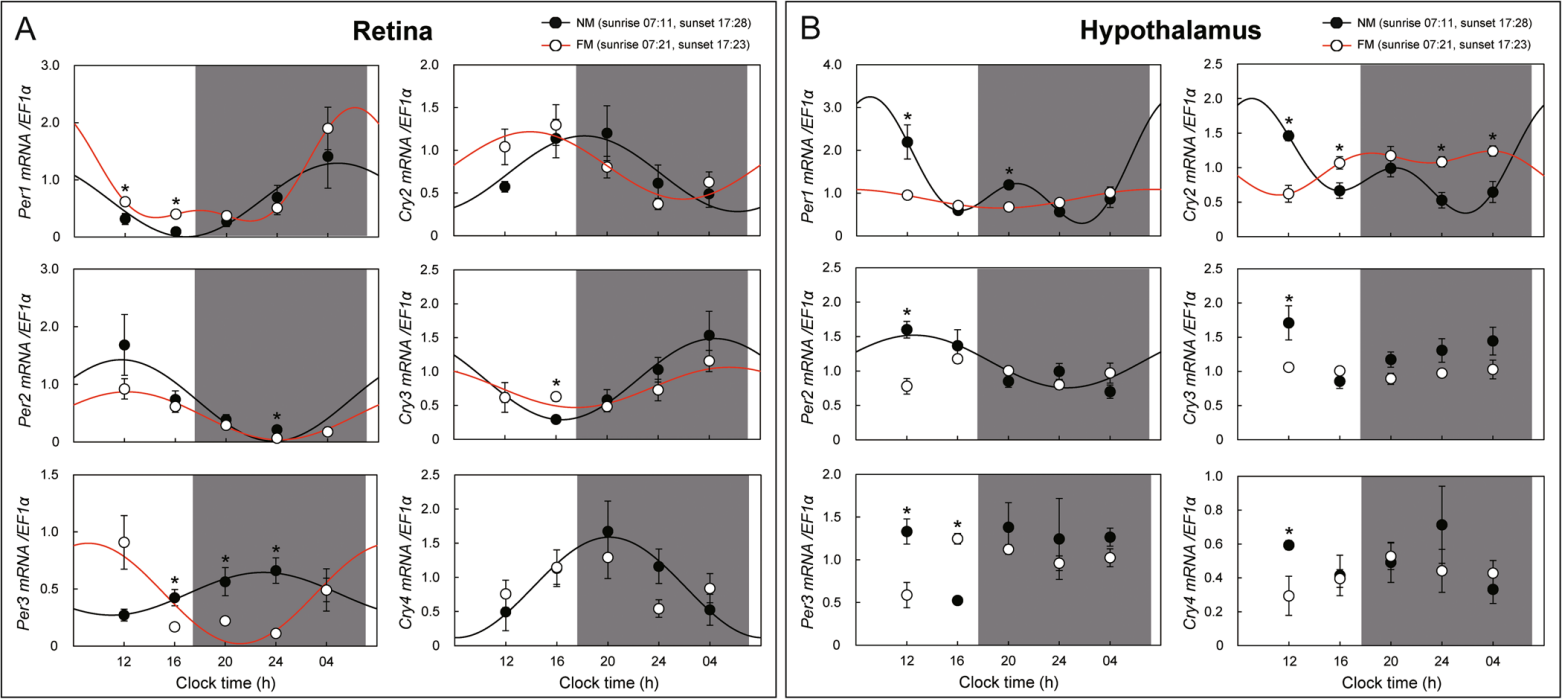
Cosinor analyses of clock gene expression levels in the (A) retina and (B) hypothalamus of the eel under natural moonlight (NM, new moon; FM, full moon). White (red line) and black (black line) represent NM and FM, respectively. *p < 0.05 at the same time point (Student's *t*‐test), mean ± SEM (*n* = 5).

In the retina, *Per1* mRNA expression peaked at 04 h and showed an oscillation in NM and FM. *Per2* mRNA expression was highest at 12 h and lowest at 24 h, showing similar oscillations between the LD and DD conditions and NM and FM conditions. *Per3* mRNA expression showed opposite oscillations in NM and FM. *Cry2* mRNA expression showed similar oscillations in NM and FM. *Cry3* mRNA expression showed the highest level at 04 hand had an oscillation in NM and FM. This oscillation was similar to the oscillation pattern observed in the LD and DD conditions. Retinal *Cry4* showed the highest level at 20 h in NM and showed an oscillation similar to that of the LD and DD conditions (Figure 8A). In the retina, *Per1*, *‐2*, *Cry3*, and *‐4* mRNA expression oscillations showed similar patterns under NM, SP, and LD conditions.

In the hypothalamus, *Per1* mRNA expression showed an oscillation in NM and FM. *Per2* showed no oscillation in FM but showed a significant difference at 12 h and showed oscillation in NM. *Cry2* mRNA expression showed opposite oscillations in NM and FM. *Per3*, *Cry3*, and *‐4* mRNA expression showed no oscillation (Figure 8B).

## 4 Discussion

In many teleosts, reproductive control is synchronized by the photoperiodic changes. However, to date, there is little evidence on the relationship between photoperiod changes and reproduction in eels. In this regard, to elucidate the correlation between changes in the photoperiod and reproduction, factors that synchronize the 24‐h cycle and photoperiod clock or light availability information for inputting day/night length information into the photoperiod clock should be analyzed. However, these input factors have not been determined for the eel. We previously confirmed that melatonin secretion in eels was inhibited by photoperiod change (SP to LP) and FM. Therefore, it is speculated that a change in the photoperiod or photostimulation of moonlight could play a key role in synchronizing the eel's biological clock. In this study, we assessed *Per1*, *‐2*, *‐3*, and *Cry2*, *‐3*, and *‐4*, which are involved in seasonal and lunar responses.

First, using RNA‐seq, we identified six core clock genes (*Per1*, *‐2*, *‐3*, and *Cry2*, *‐3*, and *‐4*, belonging to the clock gene families *Per* and *Cry*, respectively) in eels, which are orthologous genes that have been previously reported in zebrafish and other fish species. The phylogenetic relationship of each gene family was confirmed to be consistent with the results for zebrafish and other fish species. It is speculated that these can function as biological clock genes in a biological process synchronized with previously reported circadian oscillations.

However, little is known about the expression rhythms of clock genes in eels. Here, we first investigated the circadian rhythm of core clock genes in the hypothalamus and retina of pubertal male eels. Our results showed that according to the circadian cycle, *Per1*, *‐2*, and *‐3* showed more pronounced oscillations in the retina than in the hypothalamus. In the retina, in all conditions except the LL condition, *Per1* showed similar rhythmic mRNA expression, increasing just before the photophase, while *Per2* increased during the photophase. In contrast, in the hypothalamus, *Per1*, *‐2*, and *‐3* mRNA expression oscillations did not show a clear repeating pattern, and the oscillation amplitude of expression was not large compared to that in the retina. More specifically, in the retina, *Per1* mRNA increased just before the photophase, and *Per2* mRNA increased during the photophase. This was similar to the mRNA expression patterns of *Per1* and *Per2* in teleost (goldfish retina: Velarde et al. 2009; *Astatotilapia burtoni*: Song et al. 2017; turbot: Ceinos et al. 2019) and *X*. *laevis* retinas (Zhuang et al. 2000). However, since *Per1* and *‐2* mRNA maintains oscillation under DD conditions in eel, it suggests that *Per1* and *‐2* mRNA function as a key regulator of the circadian feedback loop. In this study, SP oscillations showed a clear pattern in the eel retina, but *Per3* oscillations disappeared under LP conditions, and oscillations were confirmed again under SP conditions. This is thought to be closely related to the photoperiod conditions of August to October, the spawning season of eels.

In the telencephalon and diencephalon of the Malabar grouper, in the brain of the European sea bass, and the retina of goldfish and zebrafish, *Cry2* mRNA expression peaks at the day‐to‐night transition (Yamashina et al. 2019; del Pozo et al. 2012; Velarde et al. 2009; Kobayashi et al. 2000). A similar expression was observed in pea aphids (Barberà, Collantes‐Alegre, and Martínez‐Torres 2022) and other insects, such as the bean bug and monarch butterfly (Ikeno, Numata, and Goto 2011; Merlin et al. 2013; Zhang et al. 2017). In fish, *Cry2* is a gene possibly induced by a light signal (Yamashina et al. 2019), whereas, in insects, *Cry2* does not respond to light but rather functions as a transcription factor similar to the *Per* gene (Barberà, Collantes‐Alegre, and Martínez‐Torres 2022). In this study, *Cry3* showed a similar expression pattern under all conditions and increased with a peak in the scotophase or approximately 10 h after the start of scotophase. In bony fish, *Cry3* mRNA expression peaked at midnight in the goldfish retina (Velarde et al. 2009), and oscillations were the highest at ZT24 under the 14L10D condition in the whole body and eyes of zebrafish (Kobayashi et al. 2000). In the Japanese eel, the amplitude of *Cry3* expression oscillations decreased or the phase shifted in the LL and LP conditions, respectively, when compared to those under the LD and SP conditions. This may be due to the relatively LP and exposure to high‐intensity light. However, although *Cry3* functions as a core clock gene, further research is needed to elucidate additional function of *Cry3* in the Japanese eel because of the lack of solid evidence for its ability to recognize light.

In this study, we investigated whether the presence or absence of moonlight is involved in the regulation of clock genes in eels. It is assumed that the lunar cycle or moonlight is directly related to the onset of spawning migration, vertical migration, locomotor activity, and spawning based on many previous reports on eels (Tesch 1978; Miyai et al. 2004; Tsukamoto 2006; Chow et al. 2015; Higuchi et al. 2021). Herein, the amplitude of oscillations of the core clock genes in the NM period was more distinct than that in the FM period. This is presumably because melatonin, a major regulator of clock genes, is mainly synthesized and secreted in the eel retina. We have previously demonstrated that light from the FM and transition from LP to SP inhibit melatonin secretion in eels (Hyeon et al. 2021). In particular, the level of melatonin in the eye, compared to that in the blood, increased dramatically at NM. This indicates that moonlight detected by the eye affects melatonin synthesis and secretion. Previous studies have also shown that melatonin affects the transcriptional levels of clock genes in mammals (Dardente et al. 2003; Hazlerigg et al. 2004). It has been reported that the disappearance of the melatonin signal in the retina of mice significantly affects retinal *Per1*, *‐2*, and *Bmal1* expression patterns (Hiragaki et al. 2014). This study confirmed that the presence or absence of moonlight and changes in photoperiod are involved in the oscillation of the clock genes on the core loop. Overall, the amplitude of oscillation decreased in the NM condition compared to that under the FM condition; on the contrary, in *Cry2* and *Cry3*, the amplitude decreased in FM rather than in NM. In particular, the oscillation of *Cry4* in the retina disappeared under FM conditions. This indicates that even in eels, the 24‐h retinal core clock gene is not controlled by the endogenous cyclic timer and that the normal transcription cycle of the retinal clock gene can be disrupted by light exposure time and moonlight.

Here, we for the first time investigated the expression rhythm of *Cry4* under external light conditions in eels. According to previous reports on the *Cry4* gene, *Cry4* does not have a nuclear localization signal motif or a protein‐protein interaction domain; therefore, it does not have a circadian function (Liu et al. 2015). Since *Cry4* in zebra finches is not affected by the circadian rhythm and there is no rhythm change, it is speculated that it acts as a magnetoreceptor rather than a circadian clock (Pinzon‐Rodriguez, Bensch, and Muheim 2018). In addition, strong evidence has suggested that *Cry4* does not participate in circadian transcriptional regulation as a circadian clock in zebrafish (Kobayashi et al. 2000; Liu et al. 2015), but it has been suggested that *Cry4* may serve as an upstream optical sensor expressing genes required to accompany the circadian clock in UV cones (Cermakian, 2002; Hirayama et al. 2009; Vatine et al. 2011). In this study, we confirmed that the expression pattern of clock genes in eels was altered by circadian rhythm, photoperiod, and moonlight. In particular, the amplitude of *Cry4* mRNA disappeared under the LP and FM conditions. Further research is needed to determine whether *Cry4* acts as an upstream light sensor for driving biological clock gene expression, as in the case of zebrafish, or a magnetic field receptor function according to the initiation of scattering migratory activity around their spawning migratory periods (September to October, photoperiod conversion to LP‐SP).

Previous studies have suggested that Cry4 mediates light‐dependent self‐acceptance in various animal groups (Gegear et al. 2010; Bazalova et al. 2016; Foley, Gegear, and Reppert 2011; Wiltschko and Wiltschko 2005). In particular, in seasonal migratory avians (*Erithacus rubecula*), *Cry4* is a magnetoreceptor that acts as a light‐dependent magnetic compass (Xu et al. 2021). Although the role of magnetoreceptors in fish is not well known, the existence and function of magnetoreceptors can be inferred because magnetic field recognition behaviors are observed in some fish that migrate (Shcherbakov and Fabian 2005; Takebe et al. 2012; Krylov et al. 2016; Quinn 1980; Putman et al. 2013; Cresci, De Rosa, et al. 2017; Cresci, Paris 2017; Putman 2018; Bottesch et al. 2016; Cresci et al. 2019). Eels also migrate to the Mariana Trench, near the equator, to spawn. However, unlike other migratory animals, sexual maturation begins when they start migrating. We found that *Cry4* expression in eels exhibited a similar pattern to melatonin secretion. Based on the rapid increase in melatonin secretion in mature eels in the NM, it is speculated that melatonin is related to sexual maturation (Hyeon et al. 2021).

**Figure 9:**
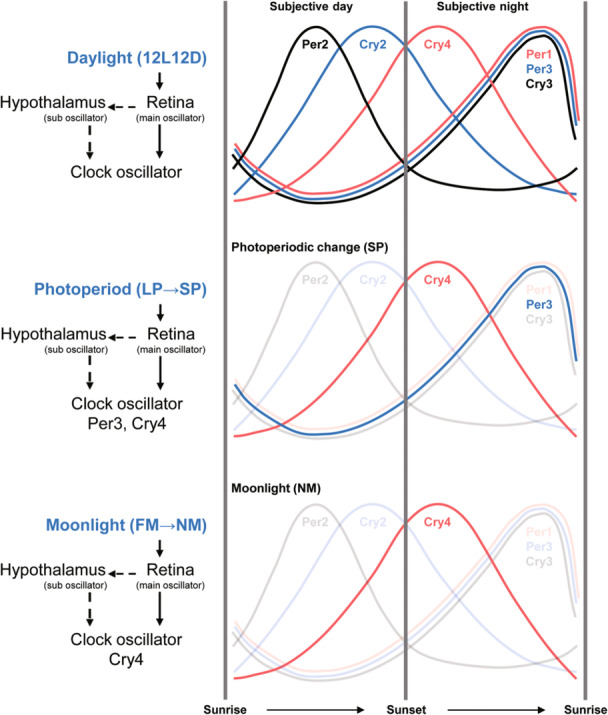
The results of this study can be summarized as follows: Light and dark responses of clock genes were observed. *Per2* mRNA expression peaked during the photophase, *Cry2* mRNA expression peaked before the onset of the scotophase, and *Cry4* mRNA expression peaked after the onset of the scotophase. *Per1*, *‐3*, and *Cry3* mRNA expression peaked before the onset of the photophase, inducing oscillations. Under the SP condition, oscillations of *Cry4* and *Per3* were induced, whereas under the NM condition, oscillations of *Cry4* were induced.

The results presented here indicate that the retina may play an important role in the circadian rhythm in eels. In addition, it was confirmed that a photoperiod change from LD to SD induces oscillations of *Per3* and *Cry4* in the retina, and the absence of moonlight induces oscillations of *Cry4* in the retina (Figure 9). This suggests that seasonal photoperiod changes and the presence or absence of moonlight affect the onset of spawning migration after puberty and that the retina, the central oscillator, provides a reproductive signal. The results of our study may help understanding the eco‐physiological aspects of sexual maturity and spawning migration in eels.

## Author Contributions

Ji‐Yeon Hyeon, Young‐Ung Choi, and Choong‐Hwan Noh performed the experiments, analyzed the data, prepared figures and/or tables, and approved the final draft. Jun‐Hwan Byun and Byeong‐Hoon Kim performed the experiments, prepared figures and/or tables, and approved the final draft. Sachithra Amarin Hettiarachchi, Jeonghoon Han, and Yuki Takeuchi analyzed the data, prepared figures and/or tables, and approved the final draft. Ji‐Yeon Hyeon conceived and designed the experiments, authored or reviewed drafts of the paper, and approved the final draft. Soo‐Youn Choi and Jong‐Eun Park authored or reviewed drafts of the paper, and approved the final draft. Sung‐Pyo Hur conceived and designed the experiments performed the experiments, analyzed the data, prepared figures and/or tables, authored or reviewed drafts of the paper, and approved the final draft.

## Ethics Statement

All procedures were approved by the Animal Care and Use Committee of the Korea Institute of Ocean Science and Technology (KIOST 2021‐0001).

## Conflicts of Interest

The authors declare there are no conflicts interests.

## Data Availability

The data that support the findings of this study are available from the corresponding author upon reasonable request.
